# Open Science in Kenya: Where Are We?

**DOI:** 10.3389/frma.2021.669675

**Published:** 2021-05-13

**Authors:** Kennedy W. Mwangi, Nyabuti Mainye, Daniel O. Ouso, Kevin Esoh, Angela W. Muraya, Charles K. Mwangi, Careen Naitore, Pauline Karega, Gilbert Kibet-Rono, Sebastian Musundi, Jennifer Mutisya, Elizabeth Mwangi, Cavin Mgawe, Silviane Miruka, Caleb K. Kibet, OpenScienceKE Collaborators

**Affiliations:** ^1^Department of Biochemistry, Jomo Kenyatta University of Agriculture and Technology, Nairobi, Kenya; ^2^Analytics Department, Africa's Talking LTD, Nairobi, Kenya; ^3^International Centre of Insect Physiology and Ecology, Nairobi, Kenya; ^4^Division of Human Genetics, University of Cape Town, Cape Town, South Africa; ^5^Center for Biotechnology and Bioinformatics, University of Nairobi, Nairobi, Kenya

**Keywords:** open science, OpenScienceKE, open access, preprints, Kenya

## Abstract

According to the United Nations Educational, Scientific, and Cultural Organization (UNESCO), Open Science is the movement to make scientific research and data accessible to all. It has great potential for advancing science. At its core, it includes (but is not limited to) open access, open data, and open research. Some of the associated advantages are promoting collaboration, sharing and reproducibility in research, and preventing the reinvention of the wheel, thus saving resources. As research becomes more globalized and its output grows exponentially, especially in data, the need for open scientific research practices is more evident — the future of modern science. This has resulted in a concerted global interest in open science uptake. Even so, barriers still exist. The formal training curriculum in most, if not all, universities in Kenya does not equip students with the knowledge and tools to subsequently practice open science in their research. Therefore, to work openly and collaboratively, there is a need for awareness and training in the use of open science tools. These have been neglected, especially in most developing countries, and remain barriers to the cause. Moreover, there is scanty research on the state of affairs regarding the practice and/or adoption of open science. Thus, we developed, through the OpenScienceKE framework, a model to narrow the gap. A sensitize-train-hack-collaborate model was applied in Nairobi, the economic and administrative capital of Kenya. Using the model, we sensitized through seminars, trained on the use of tools through workshops, applied the skills learned in training through hackathons to collaboratively answer the question on the state of open science in Kenya. While the former parts of the model had 20–50 participants, the latter part mainly involved participants with a bioinformatics background, leveraging their advanced computational skills. This model resulted in an open resource that researchers can use to publish as open access cost-effectively. Moreover, we observed a growing interest in open science practices in Kenya through literature search and data mining and that lack of awareness and skills may still hinder the adoption and practice of open science. Furthermore, at the time of the analyses, we surprisingly found that out of the 20,069 papers downloaded from BioRXiv, only 18 had Kenyan authors, a majority of which are international (16) collaborations. This may suggest poor uptake of the use of preprints among Kenyan researchers. The findings in this study highlight the state of open science in Kenya and challenges facing its adoption and practice while bringing forth possible areas for primary consideration in the campaign toward open science. It also proposes a model (sensitize-train-hack-collaborate model) that may be adopted by researchers, funders and other proponents of open science to address some of the challenges faced in promoting its adoption in Kenya.

## Introduction

According to UNESCO, Open Science is the movement to make scientific research and data accessible to all. It fosters transparency, collaboration, and accessibility in the conduct of research. A wide range of activities that reflect the concept of open science includes open data, open access publishing, open peer review, open notebook, open education, collaborative research, and citizen science (European Commission, 2017b). Engaging in these open science practices may have potential benefits to researchers, institutions, and society at large. For instance, data sharing (open data) promotes evidence-based practices and avoids duplication of effort in collecting the same datasets (Kowalczyk and Shankar, 2011), and may lead to higher citation rates for researchers (Piwowar et al., 2007; Piwowar and Vision, 2013). Open science practices may also lead to increased accountability in research, more collaborations among researchers and their institutions, and faster innovations due to sharing data and research publication (European Commission, 2017a; Ali-Khan et al., 2018).

For open science to flourish, all the stakeholders need to be involved. Some of these stakeholders include the government, research funders, academic and research institutions, researchers, libraries, ICT specialists, and the general public who use research outputs. Governments, funders, and research and academic institutions should create policies that mandate open science practices during research and create incentives to encourage the adoption of open science (Kidwell et al., 2016; European Commission, 2017b). Open science practices such as open data, open access publications, and collaborations also need to be supported by providing the necessary supporting infrastructure. These infrastructures include institutional repositories used to store electronic publications and data (Rosenblum, 2008) and National Research and Education Networks (NRENs), which support collaboration among researchers and sharing of research outputs (Dyer, 2009). Also, researchers need to be sensitized and trained on open science practices and skills regarding open access publishing, research data management, open data, and research ethics, among others (European Commission, 2017b).

In resource-constrained countries, open science faces several barriers, compared to developed economies, which hinder its adoption and growth. Among the barriers to open science include lack of policies from the governments, local funders, and institutions regarding open science practices, lack of adequate human and infrastructural capacity for training on open science, little trust in open science from researchers, and lack of awareness and skills on open science practices (Chisenga, 2018; Mwelwa et al., 2020). Notably, the lack of awareness of open science proves to be a critical barrier to open science advocacy. Even in Europe, where the European Commission advocates for policies that support increased public access to research (Gewin, 2016), the majority of researchers are unaware of the concept of open science, according to an open science survey conducted with 1,277 researchers at all career stages across Europe (European Commission, 2017b).

Open science campaign initiatives can address some of the barriers to the adoption of open science. The first step for these initiatives would be to create awareness about open science to stakeholders to ensure its positive reception. Schönbrodt demonstrates that when early career researchers are trained on open science practices, they utilize and propagate them onwards in their career paths (Schönbrodt, 2019). In Kenya, the OpenScienceKE community was founded to fill the training gap in our universities and promote open science among Biomedical students and researchers in Nairobi using the model: sensitize through seminars, train in workshops, hack through hackathons, and collaborate in an open science community. The model was an initiative through the OpenScienceKE community to ameliorate the following: lack of awareness of open science practices and tools in Kenya, low adoption of open science practices, and the absence, to our knowledge, of research describing the state of affairs in adopting open science in Kenya.

To begin with, we held awareness campaigns in various universities—this involved presentations on open science, informal surveys and discussions with participants. For the hackathons, we applied some of the skills and knowledge acquired in the workshops to assess the status of open science in Kenya through a hackathon. We employed literature search, data wrangling, and data mining to explore published literature. Although we found a growing interest in open science, the lack of awareness and skills hinders widespread adoption. Other barriers observed from discussions with different cohorts of participants were: the lack of incentives and policies in academic and research institutions to support open science, the fear of being “scooped,” and the competitive spirit within the scientific community. Additionally, we created an open resource that the students can cost-effectively publish in an open-access manner. Although this study was small and exploratory, it highlighted some insightful trends in open science in Kenya, which may be similar to other countries in Africa. Moreover, we propose a model that can be valuable for campaigns toward near-complete adoption of open science by proponents.

## Methodology

### Assessing the Status of Open Science in Kenya: Literature and Web Searches

To assess whether there are ongoing discussions on open science and its practices in Kenya, we surveyed Google Scholar for literature about open science using keyword terms: open science, open data, open source, open access, open peer review, open review, open notebooks, scientific social network, and open educational resources. We narrowed down the results to those articles that mention Kenya and the open science-related keywords in the titles. We further widened the search to articles that have Africa in the titles only. We refined the African results by excluding South Africa (excluding South Africa due to the term “Africa”): https://github.com/BioinfoNet/Status-of-OpenScienceKE-LiteratureSearch. Besides the academic articles, we conducted internet searches using the Google engine, and with the above open science-related terms, to gather information on open science in Kenya from news articles, institutional websites, podcasts, social media (Facebook, Twitter, and LinkedIn), surveys, and blogs. Further, for academic and research institutions in Kenya, we sought to assess whether they practice open science based on the following questions:

1. Does the institution have an institutional repository?
2. Does the institution have any open science policy?
3. Does the institution conduct any open science events or training?
4. Does the institution have an open educational resource?

### Assessing the Status of Open Science in Kenya: Data Mining

Since open access is a core aspect of open science, we used it as one of the metrics for assessing the adoption of open science in Kenya. We sought to address the following questions: What is the proportion of closed or open access publications with Kenyan authors? What are the publishing trends based on the journal in which the Kenyan authors published and the publication year? We used NCBI's Entrez Direct data retrieval system to query for the term “Kenya[Affiliation] AND (“1980” [EDAT]: “2020” [EDAT])” using the *easypubmed* package (Fantini, 2020) written in the R programming language (R Core Team, 2020). This search enabled us to gather about 14,488 abstracts with metadata about Kenyan authors between 1980 and 2020. We then cleaned the data using bash (GNU, 2007) command-line tools “sed” (Jargas, 2020) and “awk” (Aho et al., 1979): https://github.com/BioinfoNet/Data-mining. From the data, we assessed the proportions of closed and open access publications with Kenyan authors. For the scope of this study, Kenyan authors were taken as authors with at least an affiliation to an institution in Kenya. We also evaluated the publishing trends based on the Journal in which the Kenyan authors published, the year of publication, and author affiliations.

To understand the adoption of preprints and whether African researchers have driven the practice, we used data sourced from the PrePubMed GitHub repository (https://github.com/OmnesRes/prepub). Specifically, we sought to address the following questions: What is the number of BioRXiv preprints per country? Where does Kenya stand? What are the collaboration trends by Kenyan authors? We counted the number of preprints published by authors from each country from the BioRXiv data and analyzed for country distribution. The code used for data mining and analysis is archived at https://github.com/BioinfoNet/Data-mining.

To extract insights from the data, we used Jupyter Notebooks (Kluyver et al., 2016) to create reproducible workflows and several open-source libraries, namely: Python (Python Software Foundation, 2020), Matplotlib (Hunter, 2007), ggplot2 (Wickham, 2016), Pandas (The pandas development team, 2020), dataMaid (Petersen and Ekstrøm, 2019), to enable us to tabulate, clean and visualize the data.

### Assessing Low-Cost Open-Access Publishers

Next, we sought to investigate the cost of publishing open access and where researchers from limited-resource settings can publish in open access journals affordably. We selected publishers providing open access journals in life sciences and sought to determine whether researchers from Low-income and Low- and Middle-Income Earning Countries (LMICs) can receive exemptions, mode of eligibility, amount of waiver, and application process. Additionally, we identified preprints and predatory journals. Finally, we created a resource containing a list of publishers where students and researchers can publish open access for free or at subsidized costs (https://github.com/BioinfoNet/LowCostOpenAccess).

## Results and Discussion

### Challenges Facing Open Science in Kenya

#### Lack of Policies and Incentives From the Government, Local Funders, and Institutions

In Africa, as elsewhere in the world, open science has the potential to advance research and accelerate innovation through the sharing of knowledge. However, this requires that governments, funders, and researchers in institutions work together to realize its full potential through policies and incentives that promote open science (Mwelwa et al., 2020). We assessed whether some available policies and incentives promote open science from these research stakeholders in Kenya. We found that only a few institutions had such policies while there were no policies from the government and local funders.

Among research institutions in Kenya, the International Livestock Research Institution (ILRI) has a research data management policy and an open access policy that requires researchers to publish high-quality and reproducible science and published in peer-reviewed open access journals (International Livestock Research Institution, 2015). In universities, the University of Nairobi has an open access policy that requires all the University stakeholders to deposit their research work into the University's open repository (University of Nairobi, 2013). Jomo Kenyatta University of Agriculture and Technology has an open research data policy that promotes high research data management standards for reproducibility and sharing (Jomo Kenyatta University of Agriculture Technology, 2016). It encourages the deposition of data in its iCEOD Cloud Open Data Platform for sharing and public access. Besides these institutions, we found no other academic or research institution with formulated policies that promote open science.

Lack of policies and incentives from research stakeholders is one of the barriers to open science in Africa (Mwelwa et al., 2020). In research data management, for example, few institutions in Africa have policies in place. Without research data management policies, it is cumbersome to practice FAIR data principles (Wilkinson et al., 2016) during the research data life cycle. There has been increasing effort toward understanding research data management practices in Africa to promote good data management practices (Chigwada et al., 2017; Zotoo and Liu, 2019; Mushi et al., 2020). The research data management policies also need to focus on incentivizing researchers to share their data by considering issues that affect data sharing (Fecher et al., 2015; Kidwell et al., 2016; Ali-Khan et al., 2017). Funders should also have policies that mandate open science practices in research. For example, in South Africa, the National Research Foundation (NRF) has implemented policies that encourage open access practices in publications and data in research that it funds (National Research Foundation, 2015).

#### Limited Training on Open Science/Lack of Awareness on Open Science

Research stakeholders need to be aware of open science tools and skills to practice open science in research. In its report, the European Commission Open Science Skills Working Group recommends that researchers be sensitized and then trained on open science practices that facilitate open science such as open access, open data, open education, open peer review, and citizen science (European Commission, 2017b). In line with these recommendations, the OpenScienceKE initiative organized and ran open science awareness campaigns in major universities in Kenya, followed by a practical open access training workshop. The attendees were equipped with a variety of skills such as the use of open science tools: Git/GitHub, command line interface, application programming interface (API) for searching & fetching data, Jupyter notebooks, virtual machines, and open resource infrastructure (KENET).

Recent research indicates that the use of social media in science communication is growing and can be used by researchers as a tool to spark discussions on the issues of interest (Dong et al., 2020; Mueller-Herbst et al., 2020). We assessed the use of social media to promote open science. Although the impact has not been quantified, to our knowledge, social media has been increasingly used to promote scholarship and has been crucial in creating open science awareness (Chapman and Greenhow, 2019; Raffaghelli and Manca, 2019). A search on Twitter revealed that at the forefront of open science advocacy and practice in Kenya are the OpenScienceKE project (@OpenKE), Bioinformatics Hub of Kenya (@BioinfoHub_KE), the Kenya Education Network (@kenet_ke), Training Centre in Communication (@tccafrica), and the Eastern Africa Network for Bioinformatics Training (@EANBiT). Many other institutions and organizations within the country, not entirely dedicated to open science, maintain some prominence on social media platforms regarding open science advocacy. It may be difficult to estimate the real number of Kenyan citizens, or residents, as well as international collaborators who are actively involved in the advocacy and practice of open science in the country. However, the momentum is evident.

While we could not find a poll survey on the uptake of open science and the general practice of open science, blogs, and news articles have been published about the subject in Kenya (Juma, 2016; Wykstra, 2016). This indicates the need for more studies such as this current study to shed more light on open science in Kenya. Searches on Google Scholar using open science-related terms for any articles on the subject in Kenya only yielded 128 articles. This suggests minimal ongoing discussions about open science in Kenya. To promote further discussions about the subject of open science in Kenya, we propose the use of our model by proponents of open science, among other interventions such as Open Access (OA) week (http://www.openaccessweek.org/page/about). A search on the official websites of academic institutions indicated that among major Kenyan universities which have adopted open science practices, only the University of Nairobi (UoN) and Jomo Kenyatta University of Agriculture and Technology (JKUAT) have previously held the OA week (Table 1).

**Table 1:** Summary of different approaches to practicing open science by selected Kenyan institutions.

**Institution**	**Open access institutional repositories (IRs)**	**Open access policies**	**Training or Events**	**Infrastructure**
University of Nairobi (UoN)	Digital Repository Open Access Resource Center	UoN Open Access Policy 2012 UoN Plagiarism Policy 2013	Open Access week 2015 Open access Week 2016	Open Distance Learning Module Jomo Kenyatta Memorial Library houses KENET.
Jomo Kenyatta University of Agriculture and Technology (JKUAT)	Has an ICT Center of Excellence and Open Data with a sub-task force that advocates for various open science principles.	_	Open Data Workshop 19th Sept 2017 Open Access Workshop Open Access Week on August 18th to 24th 2010	Open and Distance Learning Modules
Kenyatta University	Has Open Access databases with links to open access journals, courseWare, and e-books Institutional repository.	_	Creating awareness about Open Science during Library Open Week in 2016	Open and Distance Learning
Strathmore University	Strathmore University Repository	_	_	_
International Livestock Research Institute (ILRI)	ILRI Repositories	Strategy on Research Publishing Research publishing procedure 6: Open Access Open Access, Intellectual Assets Data Management	Workshops and training on Open science, open access, and open data ILRI Open, Open Data Portal for access to open-source platform	_
Kenya Agricultural and Livestock Research Organization (KARLO)	KALRO Repository	_	_	_
Rift Valley Institute	_	_	_	Housing Sudan Open Archive - open access resource for all info known about Sudan
African Nazarene University	Open Access Resource	Selected Institutional Repository within the Grace Roles Library E-Resource Center for free access to open access journals	_	Institute of Open and Distance Learning
Commission for University Education (CUE) Kenya	Open Access Resource	_	_	_

“*_” indicates that no information was found concerning the practice of open science for the specified approach*.

**Figure 1:**
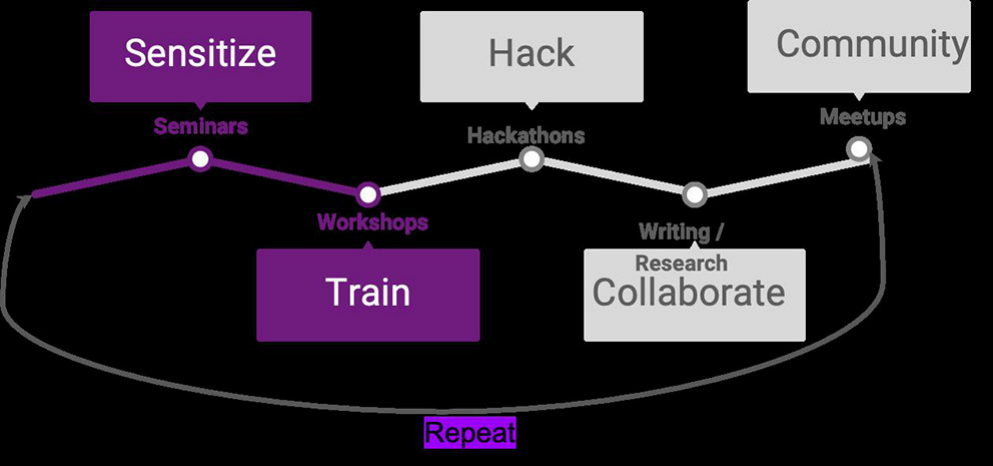
The OpenScienceKE model of promoting open science. The model involves sensitizing participants on open science practices, training them to acquire open science skills through workshops, hold hackathons to utilize the skills learned, collaborate in projects, and finally build a community of open science enthusiasts.

In considering training held within academic and research institutions, we found that JKUAT, UoN, ILRI, and Kenyatta University had held open science training events (Table 1). Training on open science is crucial to equip researchers with the necessary skills, especially, upcoming researchers, to practice open science (Schönbrodt, 2019). We believe that local initiatives such as the OpenScienceKE if sufficiently funded, can organize training episodes to equip with the necessary skills and foster communities of open science enthusiasts (Figure 1).

### The Growing Adoption of Open Science in Kenya

#### Resources to Support Open Science Are Insufficient, but Growing

##### ICT Infrastructure

The concept of openness in research demands that there is an e-infrastructure to facilitate the sharing of information. Central to this is the availability of reliable internet connection that can facilitate data sharing, especially for large datasets such as those produced in bioinformatics and imaging research. Besides reliable internet connections, storage and high-performance computing capacities are also required (Academy of Science of South Africa, 2019). In the limited-resource African setting, e-infrastructure to support open science is insufficient (Academy of Science of South Africa, 2019). However, the development of National Research and Education Networks (NRENs) in Africa is gaining momentum (Foley, 2016). NRENs support research and educational activities within a country by providing internet infrastructure and services (Dyer, 2009). The ICT infrastructure provided by NRENs is crucial in facilitating open science practices, starting with the open scholarly literature in universities and research institutions through institutional repositories. Additionally, NRENs facilitate the availability of open educational resources and enable research collaborations between local and international researchers (Foley, 2016). In Kenya, the Kenya Education Network (KENET) (https://www.kenet.or.ke/) is the NREN supporting research and education institutions. Our assessment of the adoption of the available ICT infrastructure in education and research revealed that 71 public and private universities and 21 research institutions in Kenya have memberships in KENET (Kashorda, 2020). KENET provides internet connectivity to these institutions, thus facilitating research, sharing, and teaching as well as collaboration with other institutions. NRENs such as KENET can also promote open science in academic and research institutions by providing infrastructure for open science seminars, workshops, hackathons, and any other activities concerning open science such as open sharing of data and collaboration among researchers. Indeed, the workshop and hackathon held by the OpenScienceKE initiative were supported by KENET, who provided the computing infrastructure for the training.

##### Open Educational Resources

Open Educational Resources (OERs) are teaching, learning, and research materials in any medium – digital or otherwise – that reside in the public domain or have been released under an open license that permits no-cost access, use, adaptation, and redistribution by others with no or limited restrictions (UNESCO/COL, 2012). OERs may include materials such as textbooks, videos, tests, software, full courses, modules, and other tools, material, or techniques used to assist in the access of knowledge (Van Acker et al., 2014). We assessed OERs in Kenya and found that most universities are yet to offer educational resources, which include teaching, learning, and research materials, openly and for free. One is required to be enrolled as a student to access the open learning platforms, which is a barrier for non-students. A study conducted among 798 students and 43 lecturers in 4 Kenyan universities by Pete et al. (2017) showed that awareness of OER and open licensing was low among students and lecturers, indicating a need to sensitize students and lecturers as well as other University staff on the OERs. Pete et al. (2017) found that lecturers and students would like to adopt OERs in their teaching and learning, respectively, indicating positive reception to establish the resources. Free sharing of OERs in the universities is beneficial in advancing the practice of open science in research because, in most cases, OERs are built upon published research findings. Additionally, the researchers in the institutions can use their own research findings to provide content for OERs which will, in turn, make their research open and allow for reproducibility and accountability. Importantly, free and open sharing of OERs using open licensing should be encouraged in the universities.

##### Institutional Repositories

Institutional repositories are key to providing access to literature from institutions and promoting open access practices in research. There is a growth of institutional repositories in East Africa, with Kenya leading the way (Chirwa and Ernest, 2017). Additionally, a recent study on digital research repositories in Africa showed that Kenya had 32% of repositories in Africa and was preceded by South Africa, which had 40% (Bezuidenhout et al., 2020). Kenya has several public and private universities and local and international research institutions that have embraced open research at different degrees and in different ways. We found that research institutions such as the International Centre of Insect Physiology and Ecology (*icipe*) and Kenya Agricultural and Livestock Research Organization (KALRO) have repositories where their research publications can be accessed (Table 1). Additionally, universities such as the University of Nairobi (UoN), Kenyatta University, Jomo Kenyatta University of Agriculture and Technology, and Africa Nazarene University have institutional repositories and open access resources within their digital libraries (Table 1). These resources provide access to open access journals, articles, books, databases, papers, among other resources.

Several challenges hinder the establishment of institutional repositories in Kenyan universities. These include lack of open access policies necessary for guiding researchers on sharing their work, lack of awareness of open access institutional repositories, and lack of government and funder mandates (Kakai et al., 2018).

In addition to literature repositories, some institutions such as ILRI have open access data portals where research data can be accessed. The growth of open access data repositories in African institutions has remained slow during the last decade (Onyancha, 2016). The slow growth rate may be associated with factors such as limited ICT infrastructure to manage research data, concern about data scooping upon sharing, lack of skills, among others (Nelson, 2009). Moreover, research data management services are still in their infancy in Africa (Chiware, 2020).

#### Adoption of Open Access Publishing by Kenyan Authors

**Figure 2:**
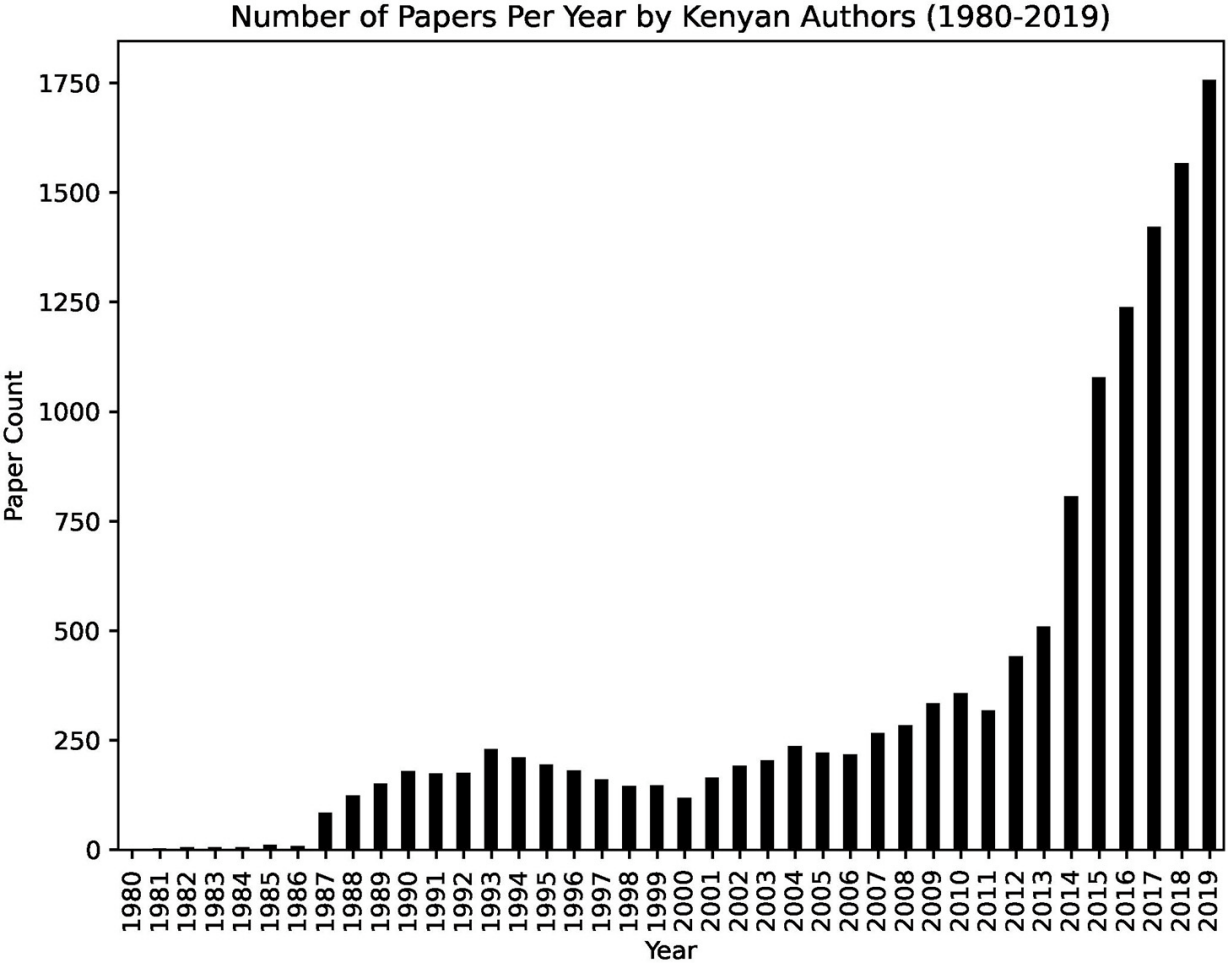
The number of papers published by Kenyan authors per year between 1980 to 2019.

**Figure 3:**
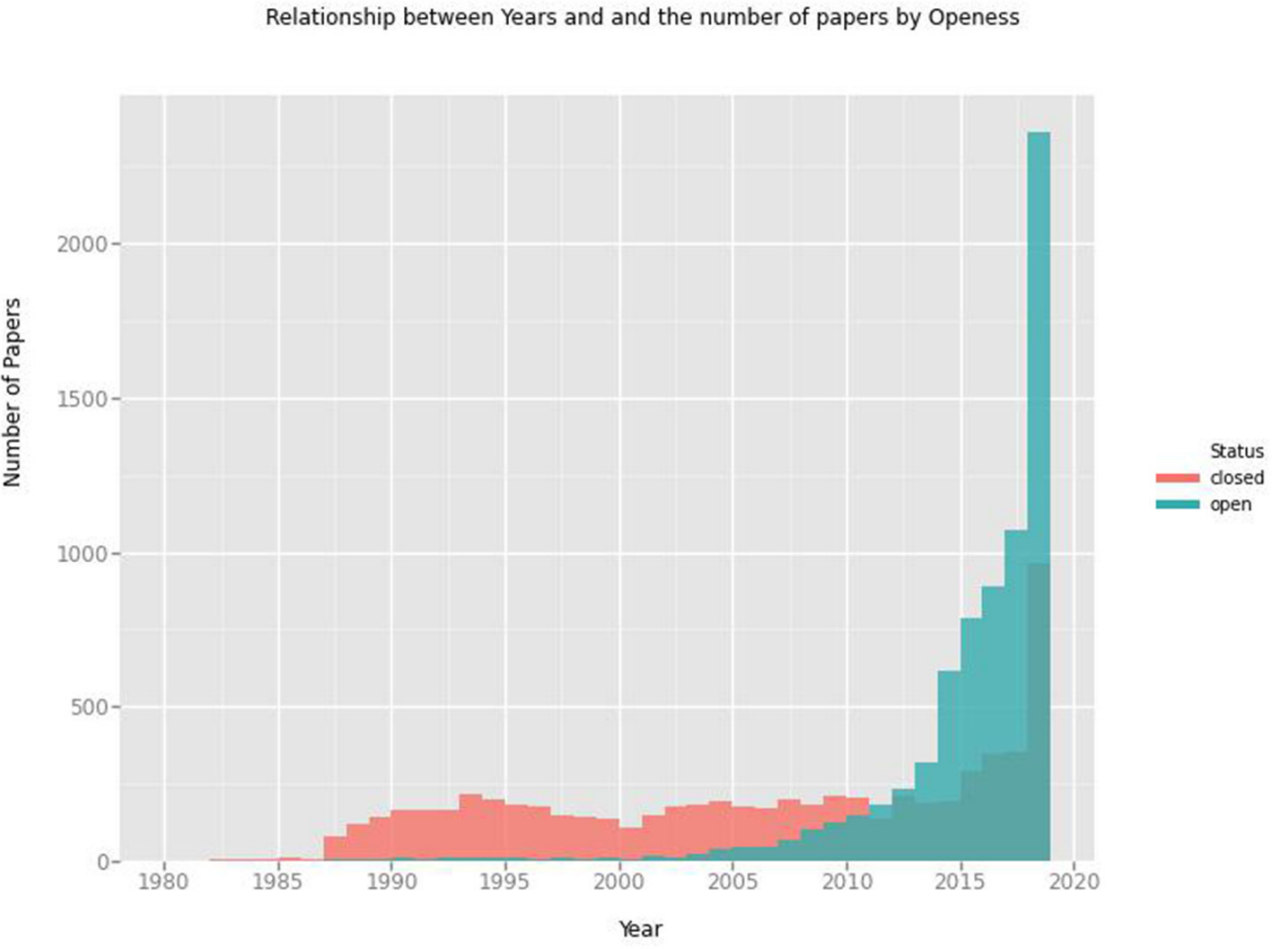
The number of papers from the year 1980 to 2020 based on whether they were open access or not. Green represents the papers that are free to access, while red represents those that are not freely accessible.

In Kenya, various stakeholders in research such as the government, academic and research institutions, libraries, students, and researchers participate in initiatives that seek to promote adoption of open access publishing (Matheka et al., 2014). These initiatives include creation and implementation of open access policies, open access repositories, and open access journals (Matheka et al., 2014). Through the existence of these initiatives, it is evident that Kenyan researchers are aware of open access publishing. The analysis of the publishing trend in Kenya shows that scholars increasingly opt to publish in open access journals. From 2012, there was an increase in the number of published papers by Kenyan authors (Figure 2) and an increase in open access publications compared to closed access publications (Figure 3), indicating a possible shift toward open access publishing. The adoption of open access publishing by Kenyan authors reflects a similar growth of open scholarly publishing across Africa (Ezema and Onyancha, 2017). The shift toward open access publishing is particularly welcome in Africa; it encourages wide dissemination of research findings in a continent where poor accessibility of scientific publications is believed to be one reason for the low research output (Tise, 2011; Raju et al., 2012).

Authors have adopted open access publishing for several reasons despite the challenges it presents. A recent study conducted among 317 scholars in public universities in Kenya by Chilimo et al. (2017) revealed that free access, larger readership, faster publication, and the desire for more citations are the primary reasons that drive open access publication. Other reasons include influence from co-authors and institutions, prestige, retention of copyright, and aligning to the funder requirements. Despite a positive trend of open access publishing, researchers still experience challenges, such as high Article Processing Charges, lack of guidelines at local administrative levels on open publishing, and the absence of an accreditation process for national journals predisposing researchers in Kenya to predatory publishers (Chilimo et al., 2017). Hence, this study recommends the establishment or improvement of robust infrastructure, capacity building, and a supportive environment for collaborative research.

#### Adoption of Preprints by Kenyan Authors

Preprints are research manuscripts deposited in a preprint server before the formal peer-review process. Publishing of preprints promotes open science and enables rapid dissemination of research findings. The use of preprints can also lead to more citations of the peer-reviewed article (Fu and Hughey, 2019). Additionally, early career researchers can benefit from preprints by getting early feedback on their work, forge opportunities for collaborations through networking, and provide an avenue for free open access publishing of their work when funds are limited (Sarabipour et al., 2019). Preprints are increasingly being adopted, particularly by researchers in life sciences (Kirkham et al., 2020). In Africa, researchers have developed the AfricArXiv preprint server, which seeks to promote and increase the visibility of African research (Ahinon et al., 2020). We sought to understand the level of adoption of pre-prints among Kenyan researchers and whether African researchers have driven the practice.

**Figure 4:**
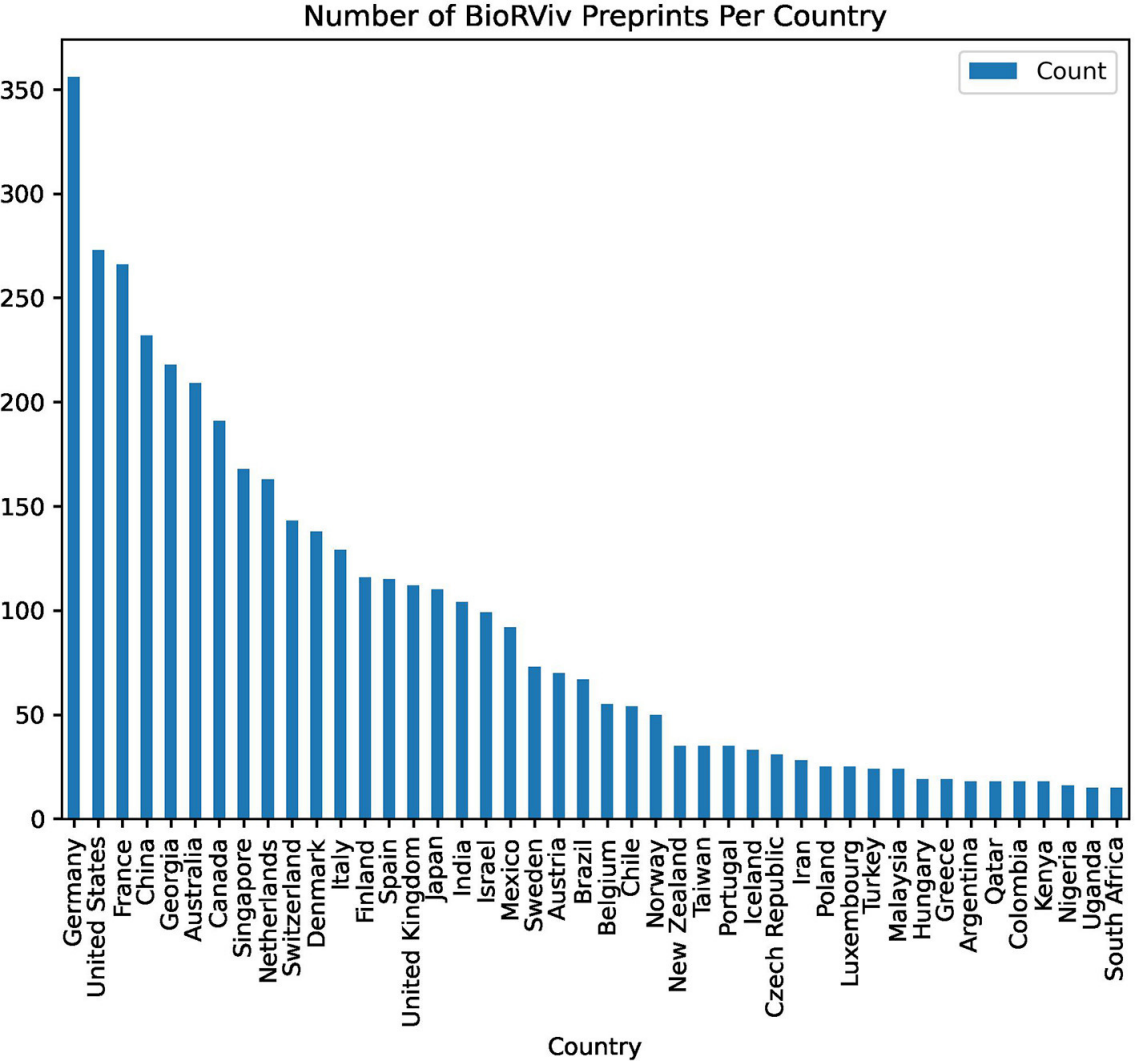
The number of BioRXiv preprint papers per country.

As of 2018, we found that 18 out of the 20,069 articles downloaded from the BioRXiv preprints (https://biorxiv.org/) had Kenyan co-author, and a majority of them (16) resulted from international collaborations. Although the data is not large, we can deduce that international collaborators drive the adoption of preprints. This trend has been observed in some African countries, including Kenya (Abdill et al., 2020). Overall, based on the 2018 data we obtained from the BioRXiv preprint server, one of the largest available, Kenya is leading Africa in preprint publications (Figure 4). However, as of 2020, South Africa and Ethiopia were leading in Africa in the number of preprints deposited in BioRXiv (Abdill et al., 2020). The growth in adoption of preprints by South African and Ethiopian researchers may be partly explained by the increased uptake of the publisher-driven opt-in options to post preprints in BioRXiv (PLOS, 2019). An assessment of authors' affiliations from the preprints as of 2018 showed that Kenyan authors mostly collaborate among themselves in their research institutions within Kenya, and there are fewer collaborations outside the country. While the nature of collaborations may be cautiously alluded to from the findings, compared with South Africa, which has more international collaboration, Kenyan researchers lead in depositing their research findings in the BioRXiv preprint server. However, more preprint servers have to be covered to get a more accurate picture of preprint adoption by countries in Africa. One difficulty we experienced in searching other preprint servers was that author affiliations were not indexed in a way that could be easily parsed.

Compared with other countries globally, the adoption of preprints in Africa still lags behind (Figure 4). Abdill et al. (2020) found that as of 2020, wealthy countries in North America and Europe contributed more preprints on BioRXiv compared to the rest of the world. South Africa was ranked in the 29th place with 182 preprints, followed by Ethiopia with 57 preprints in the 42nd place (Abdill et al., 2020). Some the reasons associated with the slow adoption of preprints include the fear of being “scooped,” lack of clear policies from publishers concerning preprints, and the misconception that preprints have low visibility and low standards of research outputs (Chiarelli et al., 2019; Sarabipour et al., 2019). Therefore, the use of preprints to disseminate research requires further sensitization and training among researchers to demystify these barriers.

### Where Can Authors Publish at a Low Cost?

The concept of openness in scientific research has long been proposed to promote equitable sharing of knowledge (Burton, 2011; Nikos, 2019). Consequently, most journals have shifted from subscription-based access to research articles and embraced open access publishing. In the latter, however, the authors have to pay article processing fees, which often run into thousands of dollars — another barrier to publishing, especially from authors in Low- and Middle-income Countries (Nabyonga-Orem et al., 2020). The lack of access to subscription-based journal articles due to their unaffordable cost hinders the accessibility of scientific literature by African researchers. However, the main barrier to equitable access and knowledge sharing in Africa is the cost of publishing, which is unaffordable for most researchers in Low- and Middle-income Countries (Nabyonga-Orem et al., 2020). For example, in most Kenyan institutions, students have to publish at least one paper at the Master's level. With limited funds and the need to publish in a reputable journal, most students experience barriers in publishing due to the high cost of article processing charges (APCs).

In this study, we observed that Kenyan authors are increasingly adopting open access publishing, consistent with observations in Low-Income countries (Iyandemye and Thomas, 2019). To encourage students and early career scientists to adopt open access, we looked into publishers that publish open access. Then, we created a resource as a guideline to publish open access, and at a low cost (https://github.com/BioinfoNet/LowCostOpenAccess). We found that most open access publishers offer waivers or subsidies to authors from developing countries. The publishers had different criteria for offering waivers. Some publishers, such as Nature Publishing Group, offered a full waiver to authors in low-income countries and a 50% discount for authors in lower-middle-income countries (LMICs). Some of the publishers link waiver policies to the corresponding author, source of funds for the research, or the financial need of the author. For example, PLoS offers waivers of about 50% or more if the research was carried out in LMICs, therefore linking their waiver policy to the financial need of authors. Publishers such as Oxford University Press and eLife offer waivers to authors in low and middle-income countries who experience financial hardships or have insufficient funds. Waivers are also available on a case-by-case basis for authors in low and middle-income countries by publishers such as Multidisciplinary Digital Publishing Institute (MDPI).

As a lower middle-income country, Kenyan authors are eligible for waivers and subsidies. We did not find any waiver model particularly targeting students. However, we noted that students are covered in the case-by-case approach, which allows them to make waiver applications as authors in financial need from developing countries. Additionally, students may also place their manuscript on preprint servers, the green route of publishing, where they can receive feedback on their work before formal publication in an open-access journal. Both the use of preprint servers (no publishing cost) and open access (with waivers) publishing will increase the accessibility of research literature in Africa.

## Conclusion

Our study suggests that there is low but increasing adoption of open science practices in Kenya. We found that there is a low adoption preprints, increasing uptake of open access publishing, limited training on open science practices, lack of policies and incentives to support open science from research stakeholders, and insufficient but growing resources to support open science. Admittedly, there is a need for an increased and concerted effort by various stakeholders for significant gains to be achieved. While the isolated effort of small advocacy groups such as OpenScienceKE and BHKi contribute to awareness and skill development, their reach is greatly constrained by a lack or limited supporting resources. To flourish, open science needs the collective effort of government policymakers, research funders, academic and research institutions, ICT specialists, and the general public who consume research outputs. We recommend that stakeholders come up with unified policies and incentives that mandate the use of open science practices in research. After formulating these policies, researchers should be sensitized and trained in their implementation in research. ICT infrastructure should also be put in place to support open science. These include institutional repositories to host digital research outputs, open educational resources, and research data storage. Moreover, researchers and institutions should also be encouraged to use NRENs and to form communities of practice to get support and offer guidance on open science practices. Although this research mainly focused on the open access aspect of open science, there is a need for larger studies on the awareness and adoption of other modes of open science practice, even to the larger geography beyond Kenya.

## Data Availability Statement

The original contributions presented in the study are included in the article/supplementary material, further inquiries can be directed to the corresponding author.

## OpenScienceKE Collaborators

Kelvin Muteru, Fredrick Musila, Ronald Tonui, Josephat Bungei, Fridah Glelis Kariuki, Rostand Chamedjeu, Esther Seroney, Mary Maranga, Rosaline Macharia, and Brian Musyoka.

## Author Contributions

KE, DO, AM, GK-R, EM, and CK devised search terms, performed internet research, and reviewed papers for assessing the status of open science in Kenya. NM, CN, CKM, SMi, and CK designed the data mining section methodology and contributed to data acquisition, data cleaning, and visualization. KM, PK, SMu, JM, CM, and CK performed internet research and created a GitHub resource for assessing low-cost open-access publishers. All authors contributed to manuscript writing, revision, and final approval.

## Conflict of Interest

NM was employed by company Africa's Talking LTD. The remaining authors declare that the research was conducted in the absence of any commercial or financial relationships that could be construed as a potential conflict of interest.
